# Resilience and Stress in Later Life: A Network Analysis Approach Depicting Complex Interactions of Resilience Resources and Stress-Related Risk Factors in Older Adults

**DOI:** 10.3389/fnbeh.2020.580969

**Published:** 2020-11-17

**Authors:** Myriam V. Thoma, Jan Höltge, Carla M. Eising, Viviane Pfluger, Shauna L. Rohner

**Affiliations:** ^1^Psychopathology and Clinical Intervention, Institute of Psychology, University of Zurich, Zurich, Switzerland; ^2^University Research Priority Program “Dynamics of Healthy Ageing”, University of Zurich, Zurich, Switzerland; ^3^Resilience Research Centre, Dalhousie University, Halifax, NS, Canada

**Keywords:** resilience resources, network analysis, early-life adversity, stress-related risk factors, later life

## Abstract

**Background:**

Emerging systemic approaches on resilience propose that a person’s or group’s adaptability to significant stress relies on a network of interdependent resources. However, little knowledge exists on systemic resilience in older survivors of early-life adversity (ELA) and how ELA affects their resource network in later life.

**Objective:**

This study investigated how ELA may be linked to the interplay of resources and stress-related risk factors in later life.

**Research Design and Methods:**

Data from *N* = 235 older adults (*M*_*age*_ = 70.43 years; 46.40% female) were assessed. Half the participants were affected by ELA through compulsory social measures and placements in childhood, and/or adolescence (“risk group”). The other half were age-matched, non-affected participants (“control group”). Using psychometric instruments, a set of resilience-supporting resources in later life and current stress indices were assessed. Regularized partial correlation networks examined the interplay of resources in both groups, whilst also considering the impact of stress.

**Results:**

Both groups demonstrated only positive resource interrelations. Although the control group showed more possible resource connections, the groups did not significantly differ in the overall strength of connections. While group-specific resource interrelations were identified, self-esteem was observed to be the most important resource for the network interconnectedness of both groups. The risk group network showed a higher vulnerability to current stress.

**Discussion and Implications:**

Network analysis is a useful approach in the examination of the complex interrelationships between resilience resources and stress-related risk factors in older adulthood.

## Introduction

While resilience research experienced an exponential growth in the last three decades, a consensus has yet to be achieved regarding how to define, conceptualize, or quantify the psychological construct of resilience (Southwick et al., 2014; Snijders et al., 2018). Nevertheless, nowadays, most experts would agree that resilience is a common and ordinary phenomenon, describing positive adaptation to negative life circumstances, and the relative stability (or swift recovery) of psychosocial, mental, and physical functioning following exceptionally stressful periods or situations (e.g., Masten, 2001; Bonanno, 2004; Bonanno et al., 2011; Southwick et al., 2014; Liu et al., 2017). Significant research interest has been placed on the identification of protective and promotive processes, and factors that underpin an individual’s resilience, such as individual characteristics, assets, and resources (Ungar, 2019).

### Traditional Conceptualizations of Resilience

In the early beginnings of resilience research (e.g., Anthony, 1974; Garmezy et al., 1984; Werner and Smith, 1992), the focus was predominantly on the identification of particular personality traits (e.g., charisma) in children (Anthony, 1974). The aim was to understand why some “invulnerable” children were seemingly unaffected by adversity (Cowen and Work, 1988), by demonstrating “…positive child development despite the exposure to multiple risk factors and adversity” (Luthar et al., 2015, p. 247). Resilience research has since come a long way beyond the investigation of outstanding personality attributes in young individuals (Masten, 2014; Southwick et al., 2014). For instance, the inclusion of a social-ecological perspective (Ungar, 2011) has broadened the scope of resilience research to the prediction of positive outcomes after exceptional stress, facilitated by a diverse set of individual (e.g., coping skills) and external (e.g., social support) psychosocial resilience factors and processes (Iacoviello and Charney, 2014; Liu et al., 2017; Snijders et al., 2018). As the concept of resilience advanced, (high) resilience came to be understood as having a meaningful and effective collection of resilience factors for overcoming a specific stressful situation (Ungar, 2011). The fundamental idea behind this definition of resilience is the assumption of accumulation, i.e., that the more resilience factors the individual possesses, the more resilient the individual (Hobfoll, 1989, 2001).

### Recent Conceptualizations of Resilience

More recent conceptualizations of resilience go beyond the assumption of accumulated resilience factors, instead emphasizing protective, and promotive processes, and factors that are mutually dependent on and influence each other (Rutter, 2012; Masten, 2014; Ungar, 2018). Within this perspective, resilience can be conceptualized as a complex network of differentially interrelated resource systems (i.e., internal systems such as biological, physiological, psychological; external systems such as social, cultural, environmental); each of which consists of resources that interact within and across systems. Depending on the stress context, the resources within an individual’s resilience network interact and can enhance or hinder each other in their ability to make an individual more or less resilient (Ungar, 2017).

### Resilience Networks

Research has only very recently begun to investigate resilience networks due to recent methodological advancements in network analysis (Costantini et al., 2015; Epskamp and Fried, 2018). Examples of such research include the examination of the interplay of resilience items and domains within a resilience questionnaire (Briganti and Linkowski, 2020), as well as the interplay of resilience, and risk factors (Fritz et al., 2018). Given the scarcity of research in this area, the latter study by Fritz et al. (2018) was used as a model upon which the current study could build. The authors applied a network analysis to examine the interrelations of various empirically supported psychosocial resilience factors (e.g., self-esteem, family cohesion) in adolescent survivors of childhood adversity and non-affected control participants. Results showed that depending on the history of childhood adversity, resilience factors were differentially interconnected with each other and their interconnectivity was further influenced by current distress. More specifically, the survivor group generally showed more negative interrelations between the resources, and their network was more negatively impacted by current distress. This suggests a deficiently functioning and vulnerable resilience network of the survivor group (Fritz et al., 2018). These findings indicate that resilience factors not only have the potential to impact each other, but can also affect, and be affected by (external) risk factors, resulting in differential outcomes.

### Gaps in the Research on Resilience Networks

In fact, the vast majority of previous studies on resilience have generally been conducted with (high-risk) children, adolescents, and to some extent, young adults. Comparatively less knowledge exists on resilience in older adulthood and to the best of the authors’ knowledge, no studies exist on the network analysis of resilience factors in older survivors of early-life adversity (ELA). In light of reports that resilience processes appear to differ between younger and older adults (e.g., Gooding et al., 2012), resilience factors identified in younger samples may not simply be assumed or adopted in research with older individuals. Given the global demographic changes towards an aging population, combined with the increasing awareness of the impact of ELA on health into old age, and the potential for resilience to shield against the negative impact of (age-related) chronic conditions (Manning et al., 2014); it is of great societal and scientific relevance to advance the understanding of resilience resources and networks in later life.

Building on the research by Fritz et al. (2018), an extensive literature search was conducted to identify psychological resilience factors in older adults and provide an empirical basis for the inclusion of resources into the network analysis of the current study. The following factors were repeatedly identified by previous studies as important in the resilience process or outcome of older individuals with experiences of (childhood) maltreatment or adversity: *Socio-economic status (SES) and SES-related resources* (Tran et al., 2013; Pietrzak et al., 2014; Martin et al., 2015; Thoma et al., 2019), *conscientiousness* (Baek et al., 2016; Thoma et al., 2019), *positive affect/emotions* (MacLeod et al., 2016; Thoma et al., 2019), *optimism* (Martin et al., 2015; MacLeod et al., 2016; Höltge et al., 2018; Thoma et al., 2019), *social support (SS) and related factors* (Pietrzak et al., 2014; Maercker et al., 2016; Beutel et al., 2017; Höltge et al., 2018; Snijders et al., 2018), *self-esteem* (Gallacher et al., 2012; Thoma et al., 2019), *self-efficacy* (Tran et al., 2013; Martin et al., 2015; Maercker et al., 2016; Höltge et al., 2018), and *self-compassion* (Höltge et al., 2019a, b; Thoma et al., 2019).

### Aims of the Current Study

It is the overarching goal of the present study to explore the structure and functioning of a resilience network, consisting of a selected set of resilience resources, in two samples of older adults with different ELA backgrounds. More specifically, this study aims to compare a network of resilience resources in a group of older adults with experiences of maltreatment and adversity within the context of child welfare practices, with that of a non-affected, age-matched control group. Furthermore, to compare the impact of stress-related risk factors on the network architecture of both groups, current stress load and stress symptoms will be included into the network models. It is commonly acknowledged that repeated and chronic stress, particularly when exposed to early in life can lead to a sensitization of the psychobiological (stress-)systems (Lupien et al., 2009). This in turn increases the vulnerability and sensitivity to future stress experiences and as such, heightens the probability for future (psycho-)pathology (McEwen, 1998; McLaughlin et al., 2010; Betz et al., 2020). It is therefore hypothesised that stress will have a differential impact on the two network models due to expected differences in the stress vulnerability of the risk and control groups. Investigating (a) the potentially different architecture of resilience, and (b) the impact of current stress on the resilience network in risk vs. non-risk individuals will help to identify key resilience resources. This could ultimately help facilitate a more efficient and resourceful targeting of protective measures in clinical interventions.

## Materials and Methods

This study was conducted at the University of Zurich, Switzerland, as part of the larger project “Differential aging trajectories in high-risk individuals with past experiences of early adversity.” The study was conducted according to the Declaration of Helsinki and was approved by the Ethics Committee of the Faculty of Arts and Social Sciences in the University of Zurich (ID: 19.4.3).

### Recruitment

Individuals with a background in child welfare practices (“risk group”) and non-affected individuals (“control group”), who were aged 50 years or older and were native Swiss German speakers, were recruited between July and December 2019. Individuals in the risk group were included if they were affected by compulsory social measures and/or placements (CSMP) in Switzerland before the age of 18 years, for a minimum duration of 1 year.

#### Risk Group

The CSMP of minors mostly entailed the placement of children and adolescents into foster care (e.g., children’s homes, foster families) or institutions (e.g., closed psychiatric or penal institutions) (The Federal Office of Justice, 2020). The CSMP practices lasted up until 1981 and originally stemmed from a welfare concept, in which local authorities aimed to shelter minors from social norm “violations” (by their parents), such as extreme poverty, single motherhood, gipsy origin, or substance addiction of one of the parents (Leuenberger and Seglias, 2008). However, in many cases the CSMP practices were implemented arbitrarily, with many families being forcefully separated by the coercive and often traumatic removal of minors from their mothers and fathers (Leuenberger and Seglias, 2008). Previous studies conducted with Swiss individuals affected by CSMP in the last century found that growing up in foster care families and institutions was commonly associated with a broad range of stress experiences, including maltreatment and adversity (for example, see Kuhlman et al., 2013). Furthermore, children in foster care often had to work hard for their living. These children often had to work full days as farm workers, which also gave them the name *Verdingkinder* or “child slaves” (Leuenberger and Seglias, 2008). In addition, they were often deprived of proper nutrition, social contact with peers, and scholarly or vocational education. Former Verdingkinder lived isolated on the margins of society, were often bullied for coming from broken families or wearing dirty clothes, and were generally considered members of the lowest social class (Leuenberger and Seglias, 2008).

Most of the participants in the risk group were recruited by the *Swiss Federal Office of Justice* (SFOJ), the office at which individuals formerly affected by CSMP up until 1981 could apply for solidarity payments. The SFOJ compiled a list of individuals who had previously agreed to be contacted for research purposes, which was given to the project lead (MVT). An information letter about the study objectives was sent to potential participants with the invitation to contact the research team in the case of interest in study participation. Some participants in the risk group were recruited by contacting individuals who were publicly available due to their active public engagement as a survivor, as well as by word-of-mouth recommendations.

#### Control Group

The recruitment of the age-matched control participants included the posting of flyers, the contacting of individuals in the sample pool of the University Research Priority Program *Dynamics of Healthy Aging* of the University of Zurich, as well as via word-of-mouth recommendations.

### Procedure

In the case of interest, potential participants contacted the study screening team. If all inclusion criteria were met, two face-to-face appointments were scheduled (lasting no longer than 2 h each), and an information package was sent out. The latter included detailed information about the study, the informed consent, and questionnaires to assess basic socio-demographic and health information. The study site (University of Zurich, their homes, other location) was chosen by the participants on the basis of their personal preferences/mobility.

Upon arrival, final open questions were answered and the informed content was signed. The first assessment (A1) then started for the risk group with an interview collecting basic information regarding their particular experiences in the context of CSMP, followed by a structured clinical interview to assess a broad range of mental disorders. With the exception of the CSMP-related assessment, the procedure of the control group paralleled that of the risk group. All interviewers were specifically trained to conduct the interviews. At the end of A1, participant were given a questionnaire package to be filled-out and brought back to the second appointment (A2), which was scheduled within 7 days. The A2 consisted of the assessment of a broad set of information on ELA and maltreatment, lifetime stress and trauma, health, well-being, functional abilities, resilience, and cognition. As with A1, A2 lasted a maximum of 2 h. At the end of A2, participants were reimbursed with 240—Swiss Francs (approximately $250).

### Instruments

A broad set of psychometric instruments were used in the larger project. Only those relevant for this study are presented in the following section, separated into instruments for risk factors, resilience resources, and outcome. Reliability statistics for all instruments and their correlations can be found in Table 1.

**TABLE 1:** Scale characteristics.

	Ω [CI]	SES	Cons	PA	OPT	SS	SeEs	SeEf	SCS	SL	SSY
Group	–	−0.33*	0.08	−0.15	−0.23*	−0.12	−0.13	−0.06	−0.11	0.24*	0.30*
SES	–	–	0.09	0.31*	0.42*	0.29*	0.40*	0.32*	0.34*	−0.53*	−0.50*
Cons	0.24^a^		–	0.17	0.09	0.14	0.21*	0.19*	0.12	−0.03	−0.07
PA	0.91 [0.89;0.92]			–	0.50*	0.36*	0.58*	0.52*	0.49*	−0.30*	−0.49*
OPT	0.75 [0.71;0.80]				–	0.40*	0.58*	0.49*	0.62*	−0.56*	−0.58*
SS	0.96 [0.95;0.96]					–	0.35*	0.32*	0.27*	−0.27*	−0.29*
SeEs	0.90 [0.88;0.92]						–	0.57*	0.75*	−0.48*	−0.56*
SeEf	0.93 [0.92;0.95]							–	0.54*	−0.32*	−0.42*
SCS	0.80 [0.76;0.83]								–	−0.46*	−0.56*
SL	0.95 [0.94;0.96]									–	0.68*
SSY	0.89 [0.87;0.91]										–

*Group, control group was set as the reference group; SES, subjective socio-economic status; Cons, conscientiousness; PA, positive affect; OPT, optimism; SS, social support; SeEs, self-esteem; SeEf, self-efficacy; SCS, self-compassion; SL, stress load; SSY, stress symptoms. Ω [CI], Omega reliability coefficient with confidence interval for ordinal scaled items. ^a^The scale for conscientiousness consists of two ordinal variables; therefore Spearman Brown coefficient was used. Correlations are adjusted for multiple testing. ^∗^p < 0.05.*

#### Risk Factors

##### Stress

To obtain an index for current stress, two self-report sub-scales of the German Stress and Coping Inventory were used (SCI, Satow, 2012). The sub-scale “total stress load” is a composite scale of the first three sub-scales (“stress due to uncertainty,” “stress due to overload,” and “stress due to loss and actual negative events”), which assess stress within the last 3 months (21 items). The sub-scale “physical and psychological stress symptoms” assessed symptoms within the last 6 months (13 items). Symptom 9 (desire for sex) was excluded in the current analysis as n = 31 participants did not answer this question (potentially due to the sensitive nature of this question for an older sample). Higher values in both sub-scales are indicative of higher stress load (potential score range: 21–147) and more physical and psychological stress symptoms (potential score range, excluding symptom 9: 12–48).

#### Resilience Resources

##### Socio-Economic Status

The *MacArthur Scale of Subjective Social Status* (Adler et al., 2000) provided an index for SES. It consists of a “ladder” (scale: 1–10) on which participants can place an “X” representing where they see themselves relative to others on the symbolic social ladder. Placing oneself on a higher step of the ladder is indicative of perceiving oneself as being closer to the highest social class (10), with respect to money, education, and occupation.

##### Conscientiousness

Conscientiousness is defined by high levels of self-control, persistence, goal-achievement, and problem-solving (McCrae and John, 1992), qualities which may be instrumental in coping with adversity and facilitating a more favorable, resilient outcome. The personality factor “conscientiousness” (Cons) was assessed with the German version of the *Big Five Inventory-10* (Rammstedt and John, 2007). Higher values in this sub-scale are indicative of a higher expression of “conscientiousness” (potential score range: 2–10).

##### Positive Affect

To assess “positive affect” (PA), the German version (Krohne et al., 1996) of the *Positive and Negative Affect Schedule* was used (Watson et al., 1988). Higher values are indicative of more PA (potential score range: 10–50).

##### Optimism

Optimism can be defined as an individual’s tendency to have favorable expectations toward future events and outcomes (Carver et al., 2010). To obtain an index for “optimism” (OPT), the German version (Glaesmer et al., 2012) of the *Life Orientation Test-Revised* was administered (Scheier et al., 1994). Higher values of the sum score are indicative of higher levels of optimism (potential score range: 0–24).

##### Social Support

To assess “social support” (SS), the German short form of the *Social Support Questionnaire* was applied (Fydrich et al., 2009). Higher values are indicative of higher perceived emotional and material SS, and higher social integration (potential score range: 14–70).

##### Self-Esteem

Self-esteem is defined as having a positive attitude toward oneself, feeling that one has good qualities, and being a person of worth (Rosenberg, 1979). The “self-esteem” (SeEs) index was obtained using the revised German version of the *Rosenberg Self-Esteem Scale* (von Collani and Herzberg, 2003). Higher scores are indicative of higher levels of self-esteem (0–30).

##### Self-Efficacy

Self-efficacy (SeEf) is defined as an individual’s belief that they are capable of coping with difficult circumstances (Schwarzer and Jerusalem, 2010). To obtain a measure for SeEf, the German version (Luszczynska et al., 2005) of the Generalized *Self-Efficacy Scale* was applied (Schwarzer and Jerusalem, 2010). Higher scores indicate higher values in SeEf (potential score range: 10–40).

##### Self-Compassion

Self-compassion (SCS) is defined as the way an individual treats themselves with warmth, compassion, and kindness in the event of failure or suffering (e.g., Raes et al., 2011). In order to assess “self-compassion” (SCS), the short form German version (Hupfeld and Ruffieux, 2011) of the *Self-Compassion Scale* was used (Raes et al., 2011). Higher values are indicative of greater levels of SCS (potential score range: 12–60).

#### Outcome

##### Satisfaction With Life

As an outcome measure, satisfaction with life (SWL) was assessed as an index of subjective well-being, using the German version (Glaesmer et al., 2011) of the *Satisfaction with Life Scale* (Diener et al., 1985). Higher scores indicate higher levels of subjective well-being (potential score range: 5–35).

### Data Analysis

Statistical analyses were conducted using R (version 3.6.0). The pre-processing of the data involved missing value analyses and checking the distribution of the model indicators. Participants missing complete scales were excluded from the analysis. Expectation maximization imputation was used for participants who had up to two items missing. A non-paranormal transformation was conducted to normalize the skewed distributions for conscientiousness, SS, and self-esteem (Liu et al., 2009).

### Network Analysis

**FIGURE 1:**
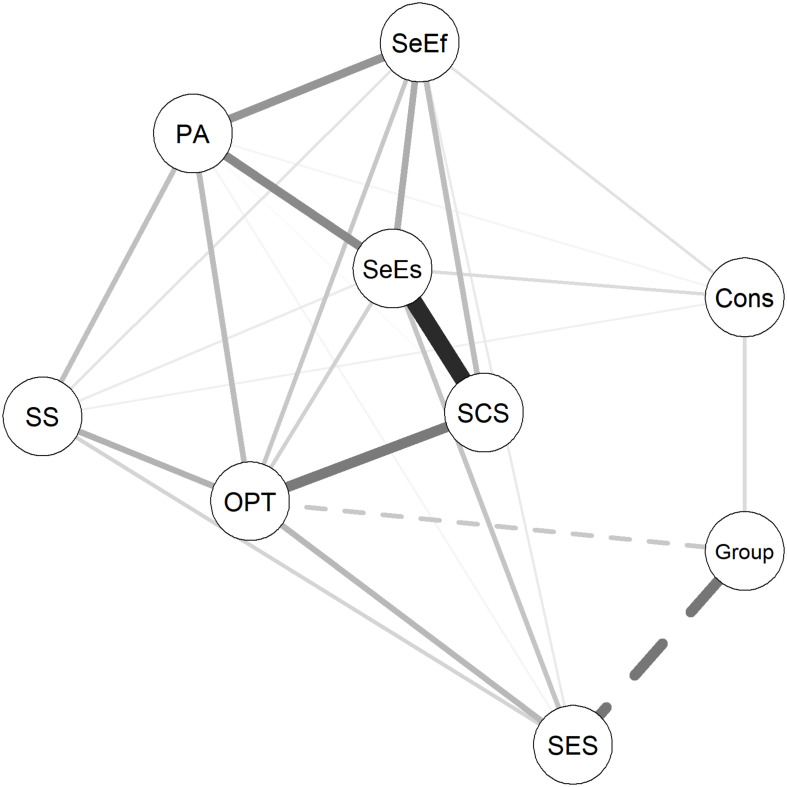
The effect of ELA on resilience resources. Group: control group was set as the reference group. Solid lines indicate positive relationships, dashed lines indicate negative relationships. The wider the line, the stronger the relationship. SES, subjective socio-economic status; Cons, conscientiousness; PA, positive affect; OPT, optimism; SS, social support; SeEs, self-esteem; SeEf, self-efficacy; SCS, self-compassion.

**FIGURE 2:**
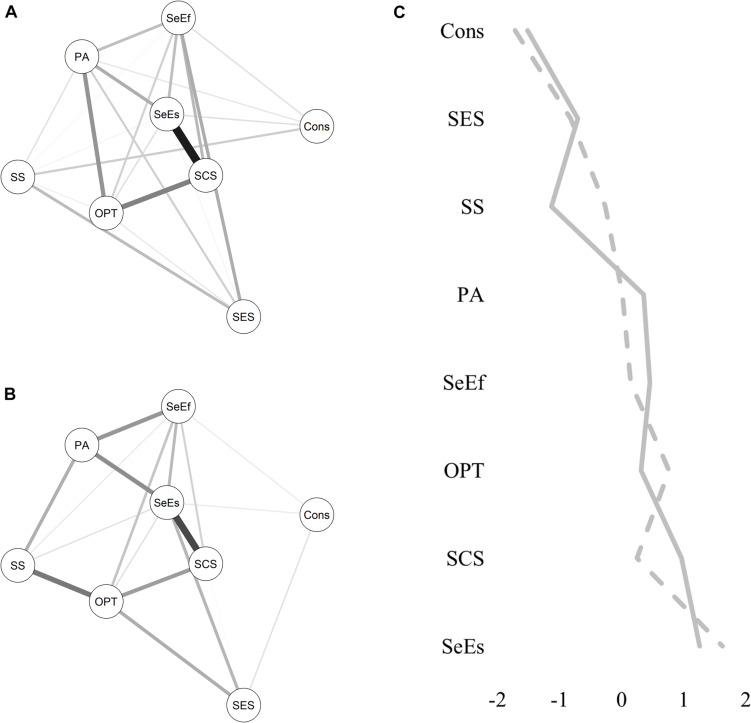
Group-specific resource networks. **(A)** Control group network. **(B)** Risk group network. Solid lines indicate positive relationships. The wider the line, the stronger the relationship. SES, subjective socio-economic status; Cons, conscientiousness; PA, positive affect; OPT, optimism; SS, social support; SeEs, self-esteem; SeEf, self-efficacy; SCS, self-compassion. **(C)** Standardized resource strength centrality: solid line indicates control group, dashed line indicates risk group. The higher and more positive the value, the higher the strength centrality of a resource.

**FIGURE 3:**
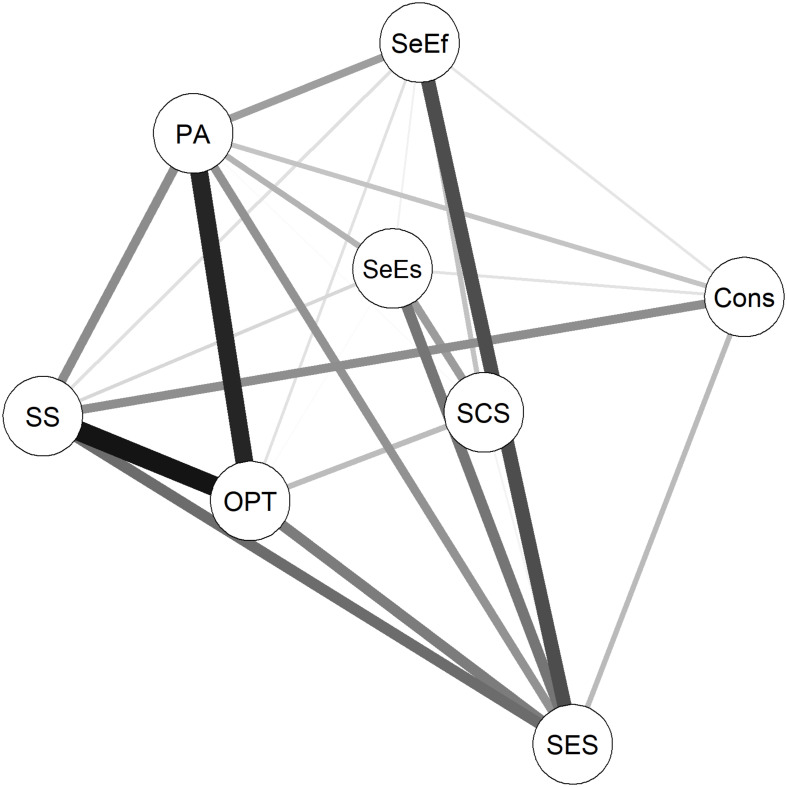
Variability network. The wider the line, the stronger the difference in edge weight between the two groups. SES, subjective socio-economic status; Cons, conscientiousness; PA, positive affect; OPT, optimism; SS, social support; SeEs, self-esteem; SeEf, self-efficacy; SCS, self-compassion.

**FIGURE 4:**
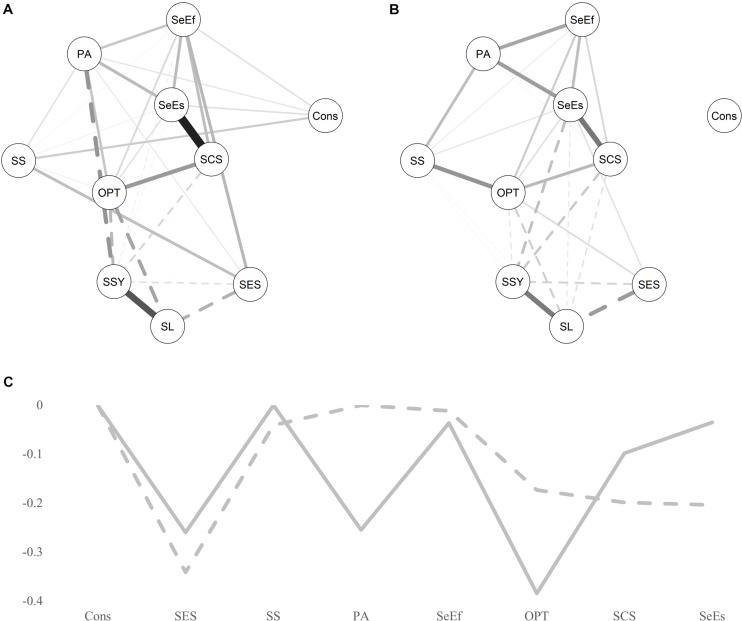
Effect of current stress on the group’s resource networks. **(A)** Control group network. **(B)** Risk group network. Solid lines indicate positive relationships, dashed lines indicate negative relationships. The wider the edge, the stronger the relationship. SES, subjective socio-economic status; Cons, conscientiousness; PA, positive affect; OPT, optimism; SS, social support; SeEs, self-esteem; SeEf, self-efficacy; SCS, self-compassion; SL, stress load; SSY, stress symptoms. **(C)** Joint negative effect of stress load and stress symptoms on resources. Solid line indicates control group, dashed line indicates risk group. The higher the negative value, the stronger the negative effect of current stress on a resource.

Overall, six network models were estimated. A network was estimated that included all resources and the group variable that indicated the experience of ELA (Figure 1). For this model, the group variable was set as 0 for the control group (indicating the reference group) and 1 for the risk group. In this case, for example, a negative relationship between the group variable and another variable in the model would indicate that ELA leads to a lower score on the other variable. Furthermore, separate resource networks were estimated for the control group and the risk group (Figures 2A,B, respectively); a variability network was estimated, indicating how much the two groups differed in their resource associations (Figure 3); and separate networks were estimated for the control group and the risk group, including all resources, as well as current stress load, and stress symptoms (Figures 4A,B, respectively).

A network analysis estimates unique relationships between all model indicators. Regularized partial correlation networks were analyzed for all six models, as these are better suited for network estimation with lower sample sizes than unregularized networks (Epskamp et al., 2017; Williams et al., 2019). A partial correlation network consists of two elements: (1) nodes, which represent the model indicators, and (2) edges, which indicate the conditional dependence between two model indicators. The networks were estimated using *bootnet* (Epskamp et al., 2018), by applying the least absolute shrinkage and selection operator with the Extended Bayesian Information Criterion (EBICglasso). This obtained parsimonious networks with meaningful edges and minimized the estimation of false positive node associations (Epskamp and Fried, 2018). EBICglasso also shrinks small and spurious edges to zero using a penalty that was set to a recommended value of 0.5 in all analyses (Epskamp and Fried, 2018).

In addition to graphically investigating specific node associations and overall patterns in the networks, several measures were investigated to compare the two groups. First, the order of node strength centrality was compared for model 2A and 2B using Pearson correlations. Strength centrality indicates the absolute interconnectivity of one node with all its connected nodes (Epskamp and Fried, 2018). Nodes with a high strength centrality have relatively many and strong associations with other nodes, and are therefore important for the structure and functioning of a network. Second, a variability network was estimated, which shows how much the edge weights differ between the two groups (for model 2A and 2B), based on each edge weight’s standard deviation across both groups (Fried et al., 2018). Third, the global strength of each entire network was compared by summing all absolute edge weights per network (overall network connectivity). This was applied to models 2A and 2B, as well as 4A and 4B, by controlling for stress load and stress symptoms (Fried et al., 2018). This facilitates three meaningful comparisons: Comparing 2A and 2B indicates which of the two groups has a weaker/stronger connected network; whilst the difference in the total strength centrality between 2A and 4A, and 2B and 4B, respectively, indicates how much each group’s network becomes negatively impacted by current stress load and symptoms. Fourth, differences in network structure and global strength between model 2A and 2B, and model 4A and 4B, were formally tested with a permutation test, using the R package *NetworkComparisonTest* (van Borkulo et al., 2017). The tests were performed with 5,000 permutations.

The R package *bootnet* (Epskamp et al., 2017) was further used to visualize the networks and to provide insight into the stability of the strength centrality estimates and accuracy of the edge weight estimates. Case-dropping subset bootstrap was used to test for strength centrality stability. To indicate sufficient stability, a correlation of at least 0.25, but better 0.50 or higher, must be estimated between the original network and the subsets (Epskamp et al., 2017). The accuracy of edge weights is indicated via bootstrapped confidence intervals (bCI). The smaller the bCI, the more accurate is the estimate. However, an edge can still be interpreted in the case of wide bCIs when analyzing a regularized network, as using EBICglasso selects the most meaningful edges. The layout of the given models is a result of the Fruchterman–Reingold algorithm, which places nodes with stronger connections closer together. For a better comparability of the models, the average layout of model 4A and 4B was used for all models.

## Results

### Sample Demographics

The samples consisted of *n* = 125 (51.2% female) for the control group and *n* = 110 (40.9% female) for the risk group. Twenty-two participants in the risk group were excluded due to missing values for complete scales. As can be seen in Table 2, the two groups significantly differed (*p* < 0.005) in their optimism, stress load, stress symptoms, and SWL. The risk group showed lower values in all resilience resources and the outcome variable SWL, and higher values in the stress indicators. The risk group also reported (non-significant) lower SS, PA, self-esteem, and SCS. Age, consciousness, and SeEf showed similar values in both groups.

**TABLE 2:** Sample characteristics.

	Control group *M* (SD)	Risk group *M* (SD)
Age	70.60 (9.68)	70.25 (12.09)
SES^a^	6	5
Cons	8.37 (1.45)	8.59 (1.38)
PA	34.10 (7.19)	31.87 (7.82)
OPT*	16.92 (4.47)	14.77 (4.45)
SS	56.32 (10.94)	53.17 (12.79)
SeEs	22.95 (4.99)	21.53 (5.38)
SeEf	29.89 (4.70)	29.29 (5.99)
SCS	41.28 (7.70)	39.64 (6.95)
SL*	33.26 (15.88)	41.83 (19.14)
SSY*	18.14 (5.41)	21.83 (6.53)
SWL*	24.95 (7.00)	21.15 (7.62)

*SES, subjective socio-economic status; Cons, conscientiousness; PA, positive affect; OPT, optimism; SS, social support; SeEs, self-esteem; SeEf, self-efficacy; SCS, self-compassion; SL, stress load; SSY, stress symptoms; SWL, satisfaction with life. ^a^Median displayed due to ordinal scale. ^∗^p < 0.005 using Mann-Whitney U Test with Benjamini Hochberg False Discovery Rate correction.*

### The Effect of ELA on Resilience Resources

Figure 1 shows which resources within the analyzed resource network are affected by the experience of ELA in the risk group. It suggests that being in the risk group is associated with a lower subjective SES and optimism (negative edges), and a higher conscientiousness (positive edge).

### Resource Network of the Control and Risk Groups

Figures 2A (control group) and 2B (risk group) show the group-specific resource networks. The connected resources show only positive associations in both groups. The strongest resource connection was estimated for SCS and self-esteem in the control group (0.55) and the risk group (0.44) (see Table 3 for the edge weights of the resource networks for both groups). Overall, the network of the control group shows more connections (51% of possible connections) than the risk group (40% of possible connections), but both show the same average edge weight of 0.09. This implies that while the two groups differ in the number of resource connections, on average, the resources are equally strongly associated when taking into account all possible resource associations in the entire network. When considering only the existing connections, the average edge weight of the control group is lower (0.13) compared to the risk group (0.16).

**TABLE 3:** Edge weights for the resource networks for both groups (model 2a and 2b).

	SES	Cons	PA	OPT	SS	SeEs	SeEf	SCS
SES	–	0.07	0	0.19	0	0.17	0	0.01
Cons	0	–	0	0	0	0.05	0.05	0
PA	0.12	0.06	–	0	0.19	0.27	0.25	0
OPT	0.05	0	0.26	–	0.32	0.07	0.14	0.23
SS	0.16	0.12	0.07	0.04	–	0.07	0.05	0
SeEs	0.03	0.08	0.19	0.08	0.03	–	0.17	0.44
SeEf	0.20	0.07	0.15	0.11	0.02	0.16	–	0.11
SCS	0	0	0	0.30	0	0.55	0.17	–

*SES, subjective socio-economic status; Cons, conscientiousness; PA, positive affect; OPT, optimism; SS, social support; SeEs, self-esteem; SeEf, self-efficacy; SCS, self-compassion. Below diagonal, edge weights for control group. Above diagonal, edge weights for risk group.*

Figure 2C shows the strength centrality profiles for both groups. The largest differences in strength centrality were found for SS and SCS: SS has a higher strength centrality in the risk group, while SCS has a higher strength centrality in the control group. In both groups, conscientiousness was the least strength-central resource and self-esteem was the strongest strength-central resource. The strength profiles of both groups showed a correlation of *r* = 0.87, indicating that the two groups show a similar ranking of the resources in terms of their strength centrality.

Figure 3 shows the variability network, which indicates how much the control group and risk group differed in their resource associations. The permutation test identified significant differences (*p* < 0.05) for the association between SS and optimism, as well as between subjective SES and SeEf. The association between PA and optimism also showed a strong, but non-significant difference between the models. As shown in Table 3, the connection between SS and optimism was stronger for the risk group than the control group. However, the connections between subjective SES and SeEf, and between PA and optimism, exist only for the control group, but not for the risk group.

Overall, the permutation test shows that the two resource networks did not significantly differ in their structure (*M* = 0.29, *p* = 0.17), or global strength (*S* = 0.13, *p* = 0.73). The global strength was 3.00 for the control group and 2.87 for the risk group.

### The Influence of Current Stress on the Resource Network of the Control and Risk Groups

The control group shows nine negative associations between the stress indicators and the resources (Figure 4A) while the risk group shows eleven negative associations (Figure 4B). Furthermore, the stress indicators show overall stronger negative associations with the resources in the control group network (−1.07), compared to the risk group (−0.97). The permutation test shows that the two resource networks (including the stress indicators) did not significantly differ in their structure (*M* = 0.26, *p* = 0.35), or global strength (*S* = 0.67, *p* = 0.09). The global strength was 4.02 for the control group and 3.36 for the risk group.

Figure 4C gives a detailed overview of how strongly the resources are affected by stress load and stress symptoms combined. The subjective SES of both groups are strongly negatively affected by stress, although the influence of stress on SES is higher in the risk group. For the control group, the strongest negative influence of stress was on optimism (−0.38), PA (−0.26), and subjective SES (−0.26). For the risk group, the strongest negative influence of stress was on subjective SES (−0.34), SCS (−0.20), and self-esteem (−0.20).

In a further step, the associations between the resources of each group were explored when controlling for the influence of current stress. In comparison to the resource-only network (models 2A and 2B), the global strength of the risk group’s resource network (in which current stress was controlled for) showed a stronger decline (a decrease of −0.81 to a global strength of 2.06) than the control group (a decrease of −0.46 to a global strength of 2.54). This indicates that current stress has a greater weakening effect on the resource network of the risk group compared to that of the control group. It is also of note that in the risk group, conscientiousness loses all its connections to other resources when controlling for current stress (comparing model 2B with model 4B), indicating that current stress likely affects the associations of conscientiousness with other resources.

### Stability and Accuracy Analyses

The graphical outputs for the stability and accuracy analyses can be found in the online Supplementary Material. The strength centrality stability analyses showed that all correlations between the original networks and their respective subsets were above the minimum requirement of 0.25, with most above 0.50. The average range of the bootstrapped CIs around the edge weight estimates ranged from 0.21 to 0.25.

## Discussion

The overall goal of this study was to compare a network of a selected set of resilience factors in two samples of older adults with varying backgrounds in early childhood adversity. Furthermore, this study aimed to investigate the impact of stress-related risk factors on the networks of the two groups. The results showed that the networks of both groups demonstrated only positive resilience resource interrelations. While the control group appeared to have a more connected network, no significant differences were observed in the network structure and global strength when compared to the risk group. In addition, although both groups showed a high level of similarity with respect to the importance of the resilience resources for the connectedness of their networks, group-specific resilience resource relationships were also identified. Furthermore, while the inclusion of current stress indices resulted in more overall negative connections in the risk group, the negative relationships observed in the control group were somewhat stronger. Finally, the results revealed that the interconnectedness of the risk groups’ resource network became weaker due to the inclusion of current stress.

### ELA and Resource Interconnectivity

As this was a cross-sectional study, the direction of influence of two model indicators is causally undirected in the network. As such, the results are interpreted by the assumption of which factor is the predictor and which is the outcome (Fried et al., 2018). Regarding the descriptive statistics, the risk group mostly showed lower levels of resilience resources, higher stress levels (stress load and stress symptoms), as well as lower well-being. This was as expected and in line with previous studies on other ELA survivors (Nurius et al., 2015; Carr et al., 2018).

The connection between group and subjective SES, and optimism was negative, and the connection between group and conscientiousness was positive. This implies that having been brought up in the context of child welfare practices (and as such, having had a higher risk for the experience of adversity and maltreatment), may be associated with lower subjective SES and optimism in later life. This is in line with previous research conducted with comparable samples of individuals affected by child welfare practices in other countries in the last century (e.g., Sigal et al., 2003; Kuhlman et al., 2013; Lueger-Schuster et al., 2018; Carr et al., 2019). The finding that the risk group was linked to lower levels of optimism in the current study is also supported by previous research, which found a negative relationship between childhood emotional maltreatment and dispositional optimism in older adulthood (Broekhof et al., 2015). The positive connection observed between a background in childhood welfare practices and conscientiousness was unexpected and may provide tentative evidence for stress-related resilience in this sample. Results also showed that although the network of the risk group had fewer connections, the overall strength of connections and network structure did not significantly differ in comparison to the control group. Given that the risk group reported significantly higher stress levels (stress load, physical, and mental stress symptoms) and lower levels of well-being, these findings may suggest that having more, though somewhat weaker connections is characteristic of a better functioning resource network than having few strong connections.

The network analyses further revealed that the resource networks of both the risk and control groups showed only positive interrelations. This finding contrasts with a previous network analysis study on resilience and childhood adversity in adolescents, which identified negative interrelations between several resilience factors (Fritz et al., 2018). One explanation may be related to the differing ages of the investigated samples. It may be that in an older sample, individuals have had more experience across the life course with successfully utilizing and strengthening their resource network, which may in turn help to buffer against the impact of ELA. However, to make more concrete conclusions, future research should investigate the impact of adversity on resilience resource networks at varying stages across the life span.

Both groups also showed a similar strength centrality profile, indicating that roughly the same resources were important for the functioning of both networks. Self-esteem was the most important resilience factor for both groups’ network interconnectedness. As such, self-esteem may be an optimal resilience resource upon which to focus to facilitate an efficient targeting of protective measures and clinical interventions for older adults dealing with the negative effects of ELA. Conscientiousness, on the other hand, showed the least relationships with other resilience resources. Thus, while its positive connections may suggest a protective influence, its reduced interrelatedness with other resilience resources imply that conscientiousness may be less important for consideration as a primary target of resilience interventions.

In relation to edge weights, the strongest differences were found between SS and optimism, PA, and optimism, as well as SeEf, and subjective SES. The connection between SS and optimism was stronger for the risk group than the control group. Adversity experiences in an individual’s early environment can shape their expectations regarding affect regulation, social interactions, support availability, and help-seeking behaviors (Riggs, 2010; Lee et al., 2015). Thus, it may be that due to higher levels of interpersonally experienced adversity in their childhood and adolescence, individuals in the risk group place a higher value on SS in their adult life. In support of this, previous research with a similar sample of Swiss Verdingkinder found that SS predicted resilience in later life (Maercker et al., 2016). This finding is further supported by the observed connection with optimism in the current study, which has been shown in the literature to be associated with the capacity to seek and utilize SS (Carver and Scheier, 2014). Therefore, in facilitating more resilient outcomes, survivors of ELA in welfare-contexts may uniquely benefit by drawing upon the resilience resources of optimism and SS.

The control group additionally demonstrated positive connections between PA and optimism, as well as SeEf and subjective SES; connections that were not observed in the risk group. It may be that given their background in ELA, individuals in the risk group are less able than the control group to engage with internal resilience resources that are reliant on the self, such as PA or SeEf. In support of this, recent research with a similar sample of *N* = 220 of adult survivors of institutional ELA in Austria found that institutional childhood abuse predicted lower levels of SeEf and self-esteem in adulthood. The study concluded that prolonged exposure to ELA in such institutional welfare settings may lead to reduced self-beliefs and beliefs in one’s ability to succeed in difficult situations (Weindl et al., 2018). However, given the novelty of the present findings, additional longitudinal, group-comparison research is needed to explore this further.

### Current Stress and Resource Interconnectivity

A critical facet of resilience research is the examination of the interplay between risk factors and resilience (Windle, 2011). In the current study, the risk factors of current stress load and stress symptoms were introduced into the resilience resources network. The resource network of the risk group appeared to be more vulnerable to current stressors, as indicated by a stronger decline in overall resource interconnectivity. This in line with the findings of Fritz and colleagues, which identified a more dysfunctional resilience network in the adolescent survivors of childhood adversity when controlling for the influence of current stress (Fritz et al., 2018). However, in the current study, although more negative relationships were observed between the stress-related risk factors and resilience resources in the risk group, the strength of the negative connections was stronger in the control group. Within the risk group, the resilience resources most severely affected by current stress were those related to the self, such as SCS, subjective SES, and self-esteem. This contrasts with the network of the control group, in which current stress more severely affected the resilience resources linked to positivity, i.e., PA and optimism. While the influence of stress on positivity may be an expected finding (e.g., Schilling and Diehl, 2014; Horiuchi et al., 2018), the impact on the self in survivors of child welfare adversity is of particular interest. It may be that individuals who experienced child adversity and degradation in these welfare contexts have a vulnerable self-perception and are more susceptible to the impact of current stress. This may highlight potential targets for intervention, such as improved self-perception, SCS, and self-esteem. However, given the lack of causality in the data, additional research should further investigate this novel finding.

### Strenghts

This is the first study to apply network analysis in the investigation of resilience resources and their interplay with stress, in a sample of older survivors of ELA experienced within the context of child welfare practises. Previous research using network analysis to examine resilience networks has thus far only assessed children and adolescents (e.g., Fritz et al., 2018). By examining adults and older adults, this study expands the literature on resilience resource networks into older life stages. In addition, the application of a dynamic resilience conceptualization allowed for the modeling of a complex network of differentially interrelated internal and external resource systems. While future (longitudinal) research is need to replicate these findings, the identification of a positive network of resilience resources may be beneficial in highlighting potential targets for clinical intervention with adult survivors of ELA. An additional strength was the use of an age-matched control group not affected by welfare-related adversity in childhood. This allowed for a comparison of the resilience resource networks and interpretations to be made specific to adult survivors of ELA in the context of welfare practices. Furthermore, using network analysis in the realm of resilience research adds another crucial perspective to the characteristics of resources and resilience interventions: rather than identifying only the most effective resources in a stressful context, network analysis provides the opportunity to identify the central resources. Central resources are important for the sustainability of a network and are a potential target to efficiently influence other resources.

## Limitations

Several limitations warrant consideration when interpreting the results of this study. This study used a cross-sectional design, which hinders the determination of a causal relationship between the model indicators. Resilience is best assessed using a longitudinal approach to capture its dynamic nature (Snijders et al., 2018), as resilience can develop and change over time in response to different stressful contexts (Luthar et al., 2000). Furthermore, the sample size was relatively small, which limited the power and scope of the analysis. For instance, small homogeneous groups can lead to a low differential variability, which can make it less likely to detect resource connections within a network (e.g., Fried and Nesse, 2014). Also, due to the context-specificity of resilience (Ungar, 2011), even the networks between institutionalized children and children who lived with foster families might differ, which could not be tested in the current study due to the limited sample size. Related to this, the ELA of this historic risk sample, i.e., being raised in the context of welfare practices, is a rather specific form of adversity and may hinder the generalizability of the findings. As such, this study should be replicated across larger samples and within differing ELA contexts in order to empirically test the generalizability of the network structure. Furthermore, although this study assessed psychological and social resources in older age, additional contextual resources for resilience could be added in a next step. For instance, an ecological systems’ approach to resilience would warrant the addition of socio-ecological resources, such as community, cultural, or economic resources (Ungar, 2018).

## Conclusion

To the best of the authors’ knowledge, this was the first study to apply network analysis to explore the interplay of resilience and risk factors in two age-matched, older samples with differing backgrounds in ELA. Although the network model approach is still a comparably young perspective in the field of psychopathology, and resilience research in particular (see Fried and Cramer, 2017 for challenges of the network perspective); this study has shown that it is a suitable methodology for the examination of the interrelationships between resilience and risk factors. The findings of the current study identified a complex network of resilience resources, highlighting resources that were more strongly connected in the separate resilience networks of both the risk and control groups. It further examined the interplay between resilience resources and risk factors (i.e., current stress) and demonstrated group-specific changes in the resilience networks following the introduction of the risk factors. Despite the difficulties with causal interpretation of findings, network analysis is a useful tool for moving forward resilience research by providing essential steps toward a better understanding of the complex construct of resilience.

## Data Availability Statement

Due to the sensitive nature of the data, the data cannot be published on a public data repository. The raw data will instead be held in the university archives in accordance with the ethical regulations. Requests to access the datasets should be directed to m.thoma@psychologie.uzh.ch; jan.holtge@dal.ca.

## Ethics Statement

The studies involving human participants were reviewed and approved by the Ethics Committee of the Faculty of Arts and Social Sciences in the University of Zurich (ID: 19.4.3). The patients/participants provided their written informed consent to participate in this study.

## Author Contributions

MT, JH, and SR conceived the idea for the study and were responsible for the conception and design of the study. MT and SR were managing data collection. CE and VP were involved in data collection. JH conducted all data analysis. MT and JH wrote the manuscript together-both contributed equally to the manuscript. SR profoundly contributed to the writing of the manuscript and the interpretation of the data. SR proofread the manuscript. All authors critically revised the manuscript before submission.

## Conflict of Interest

The authors declare that the research was conducted in the absence of any commercial or financial relationships that could be construed as a potential conflict of interest.
