# Supplementary material for: Resilience and Stress in Later Life: A Network Analysis Approach Depicting Complex Interactions of Resilience Resources and Stress-Related Risk Factors in Older Adults
Source: Front Behav Neurosci. 2020 Nov 17;14:580969. doi: 10.3389/fnbeh.2020.580969 (PMC7705246; doi:10.3389/fnbeh.2020.580969)
Supplement: Supplementary file 1 [file Table_1.DOCX]

**Supplementary Material**

**Edge weight accuracy and centrality stability**

1. Edge weight accuracy and centrality stability for model 1
2. Edge weight accuracy and centrality stability for model 2A
3. Edge weight accuracy and centrality stability for model 2B
4. Edge weight accuracy and centrality stability for model 4A
5. Edge weight accuracy and centrality stability for model 4B

**Note:** SES: subjective socio-economic status, Cons: conscientiousness, PA: positive affect, OPT: optimism, SS: social support, SeEs: self-esteem, SeEf: self-efficacy, SCS: self-compassion, SL: stress load, SSY: stress symptoms.

1. Edge weight accuracy (left) and centrality stability (right) for model 1: Resource network including group variable.


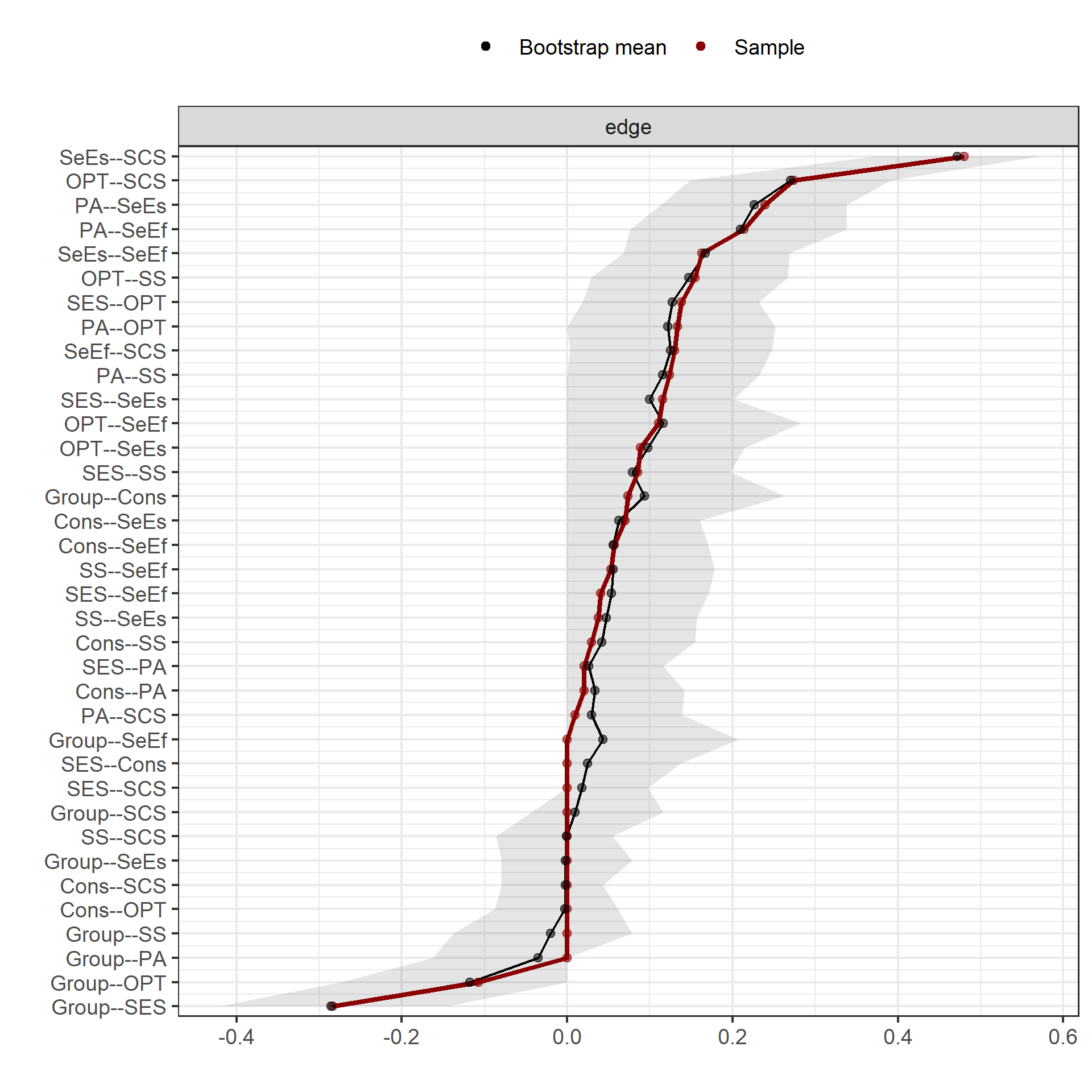

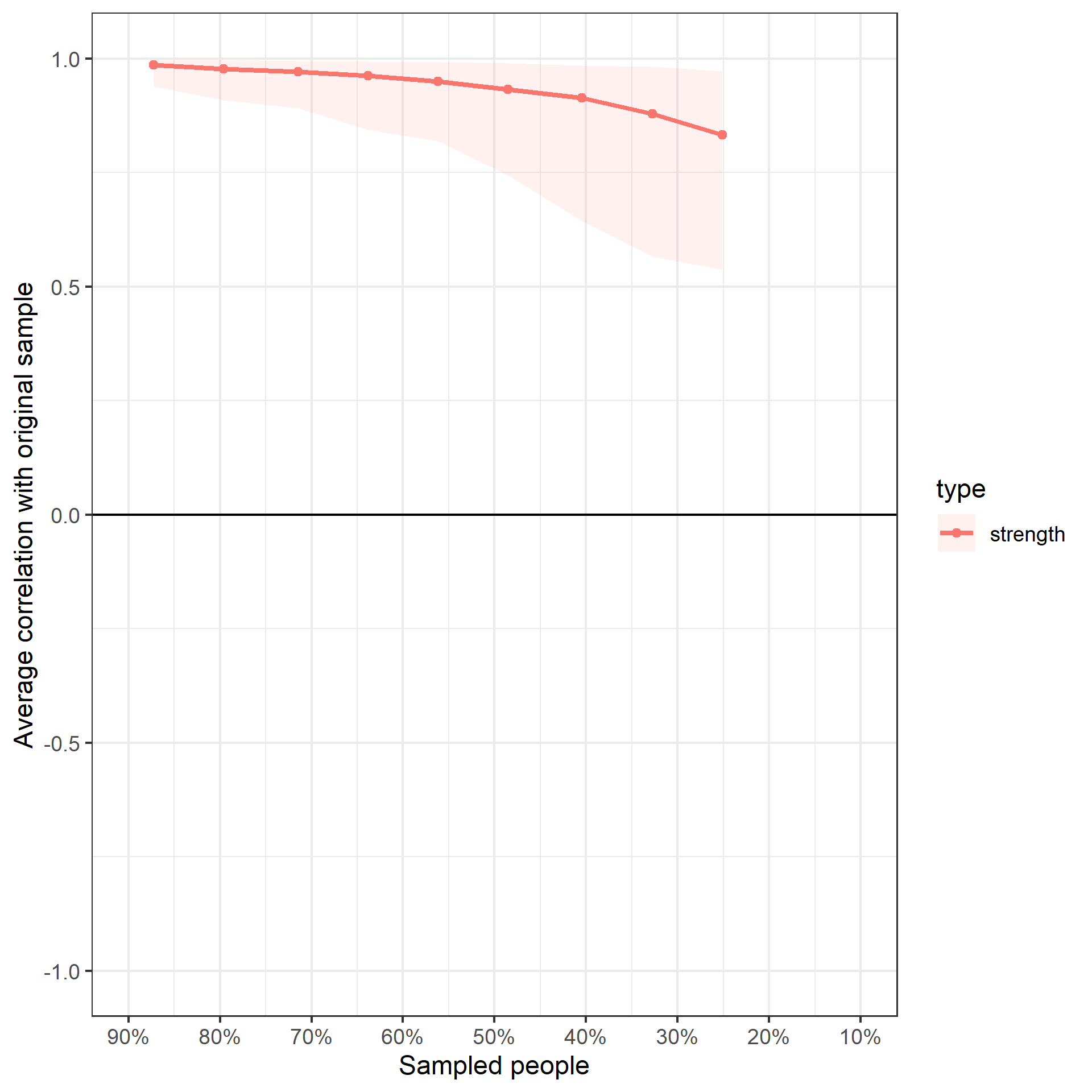


CS-coefficient: .67

1. Edge weight accuracy (left) and centrality stability (right) for model 2A: Resource network of control group.


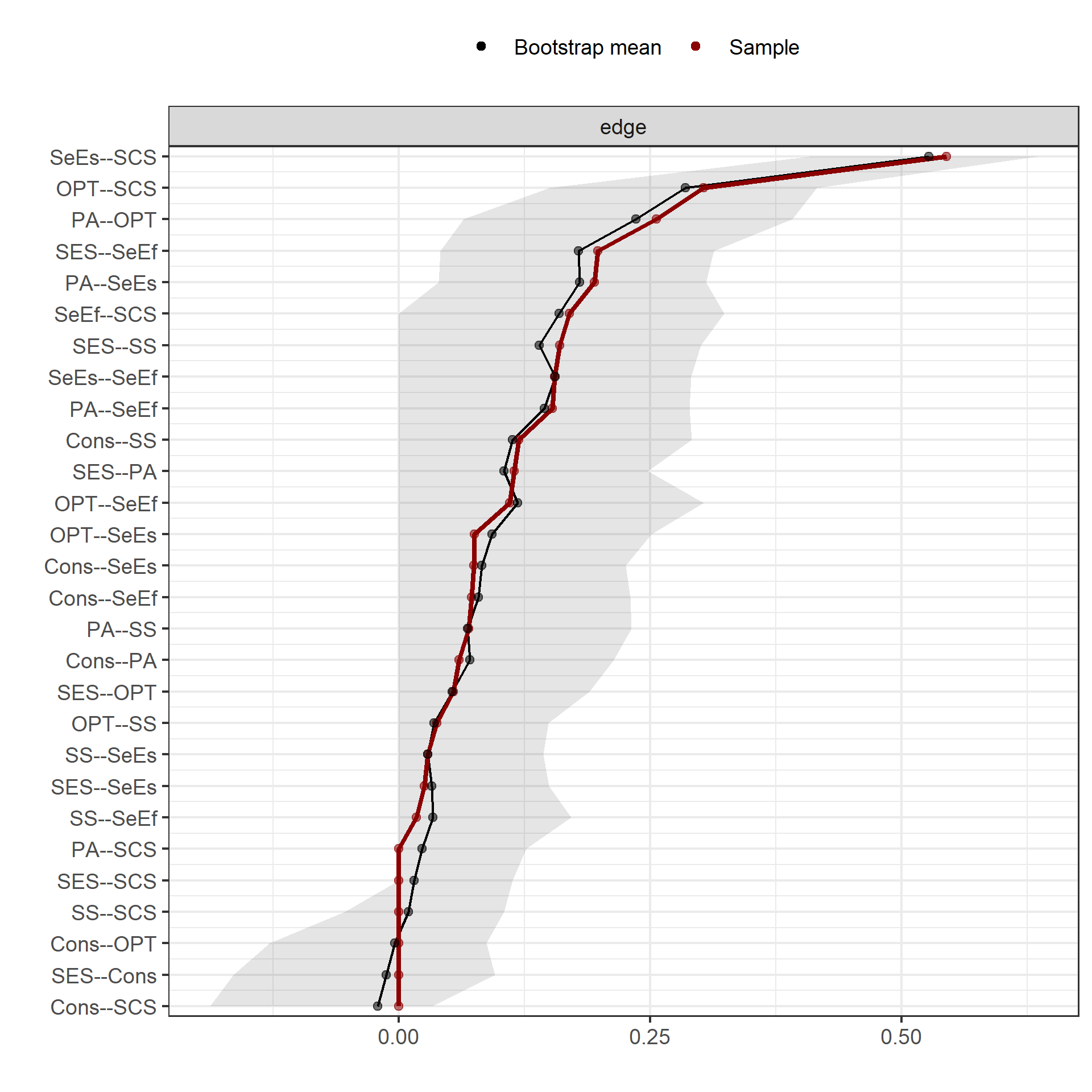

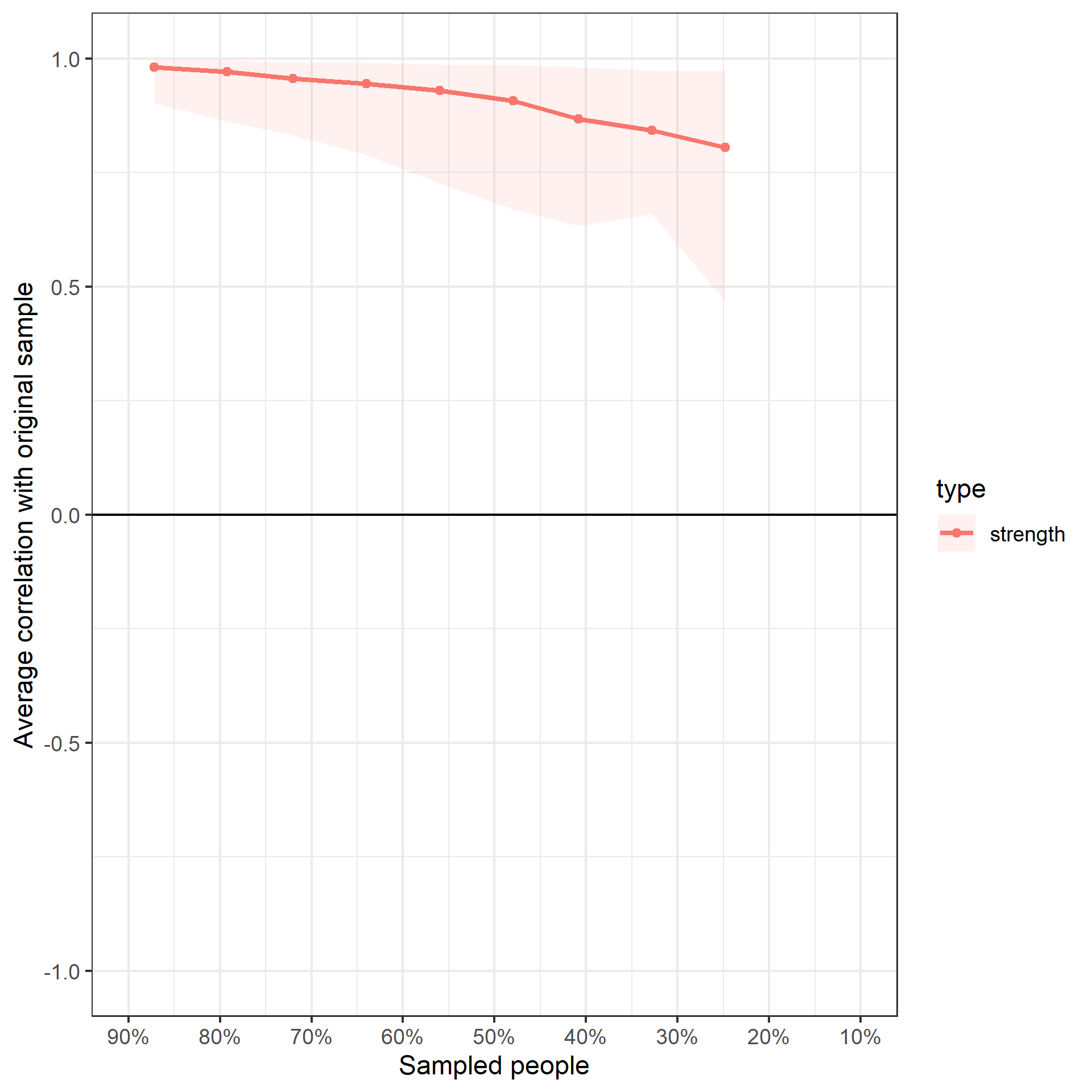


CS-coefficient: .59

1. Edge weight accuracy (left) and centrality stability (right) for model 2B: Resource network of risk group.


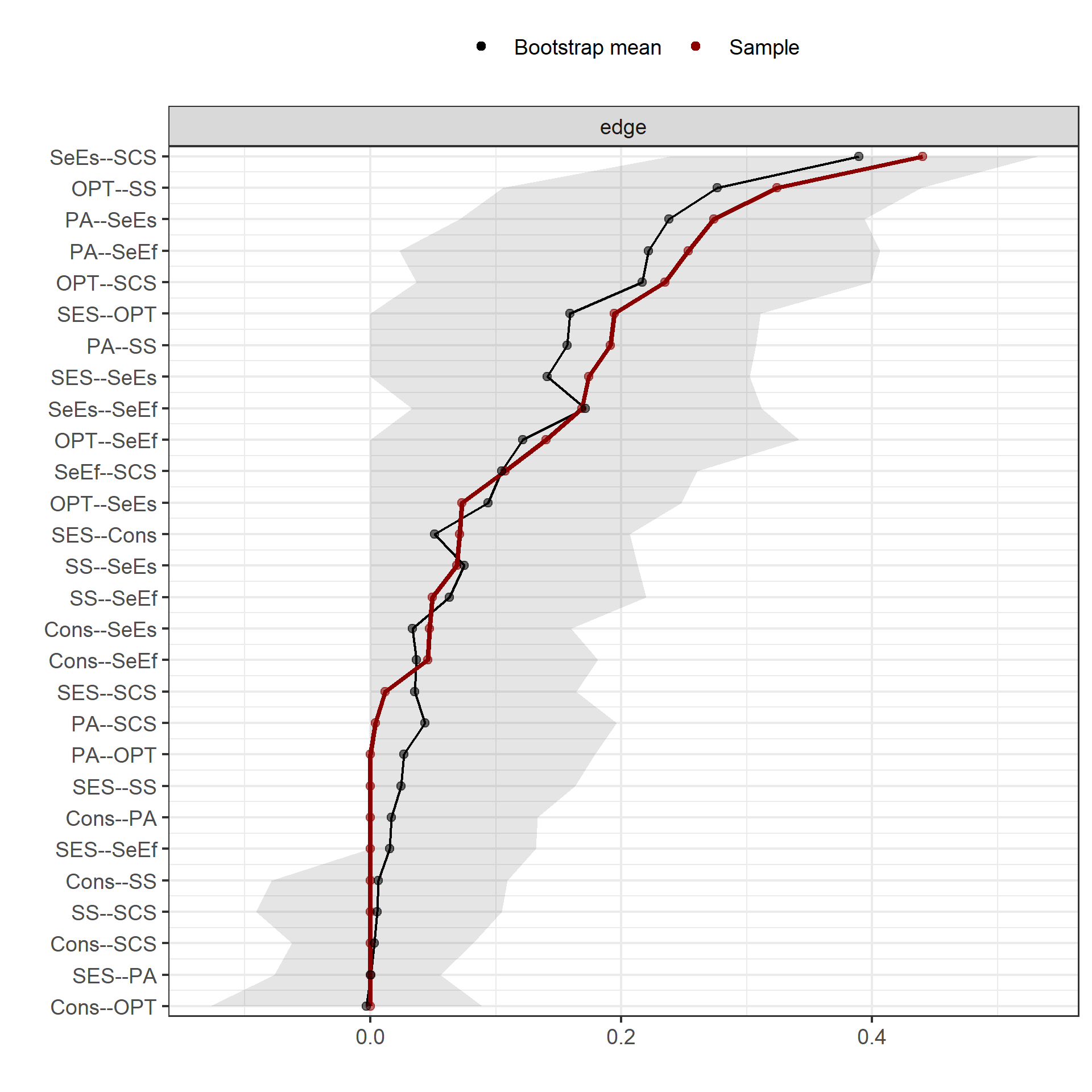

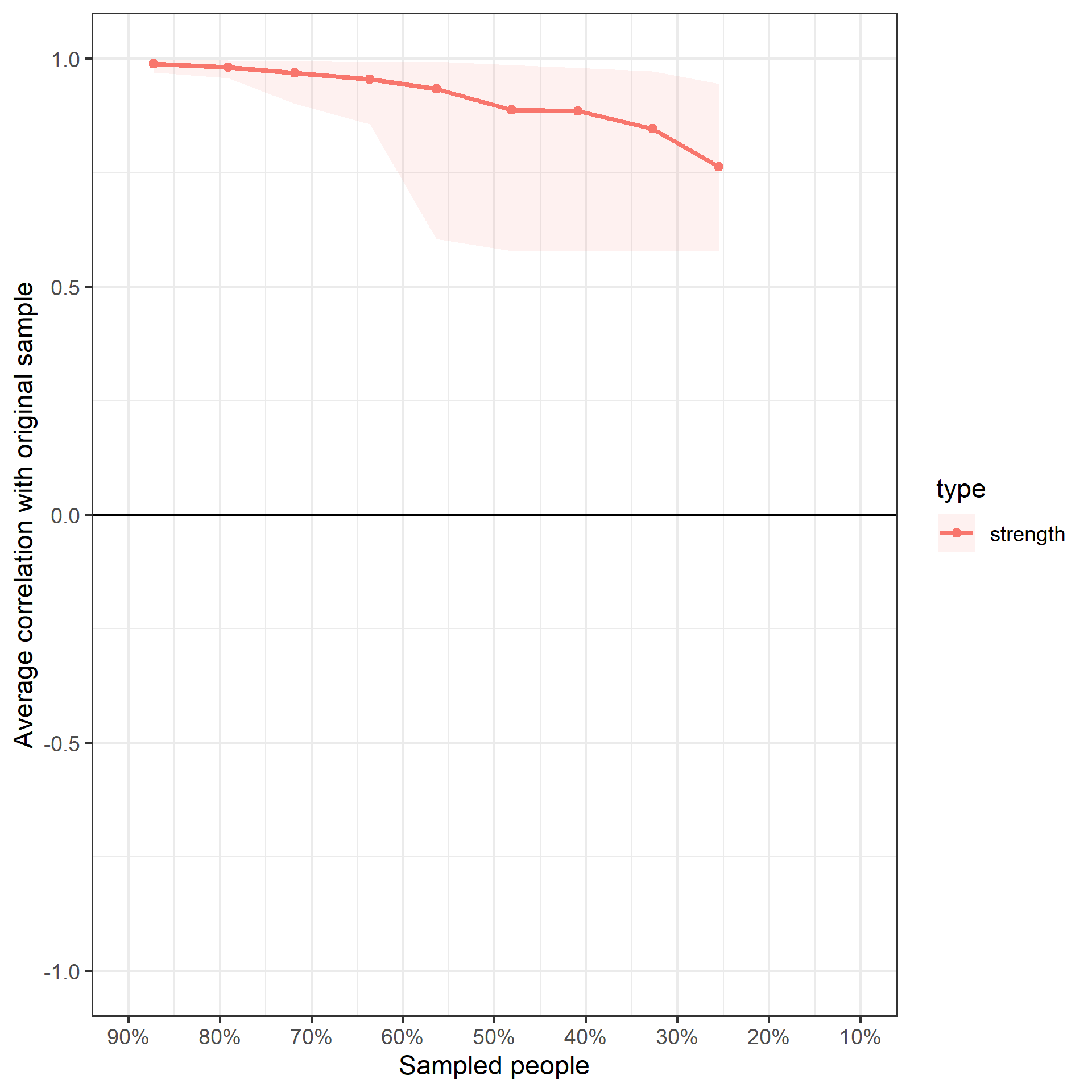


CS-coefficient: .36

1. Edge weight accuracy (left) and centrality stability (right) for model 4A: Resource network of control group including stress indicators.


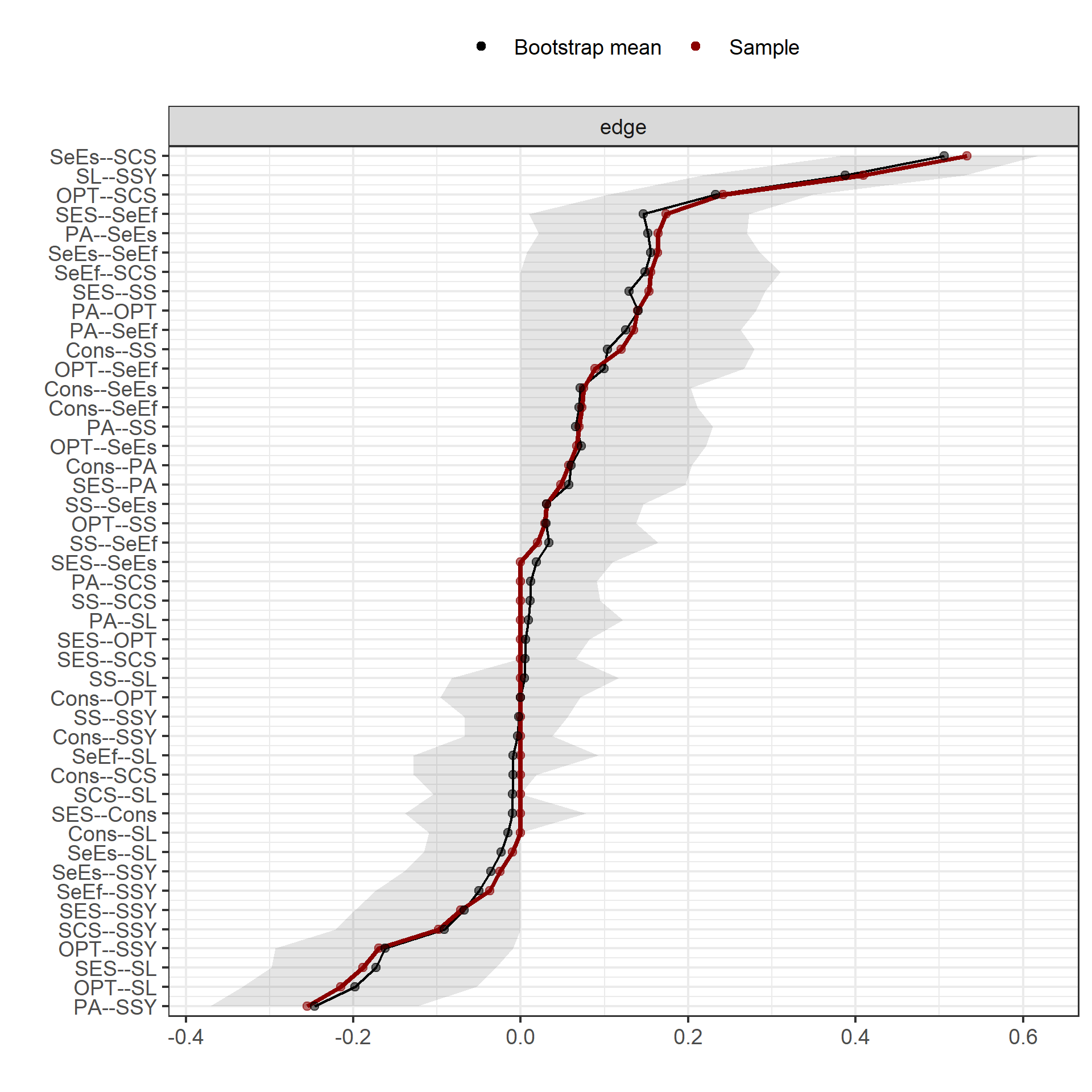

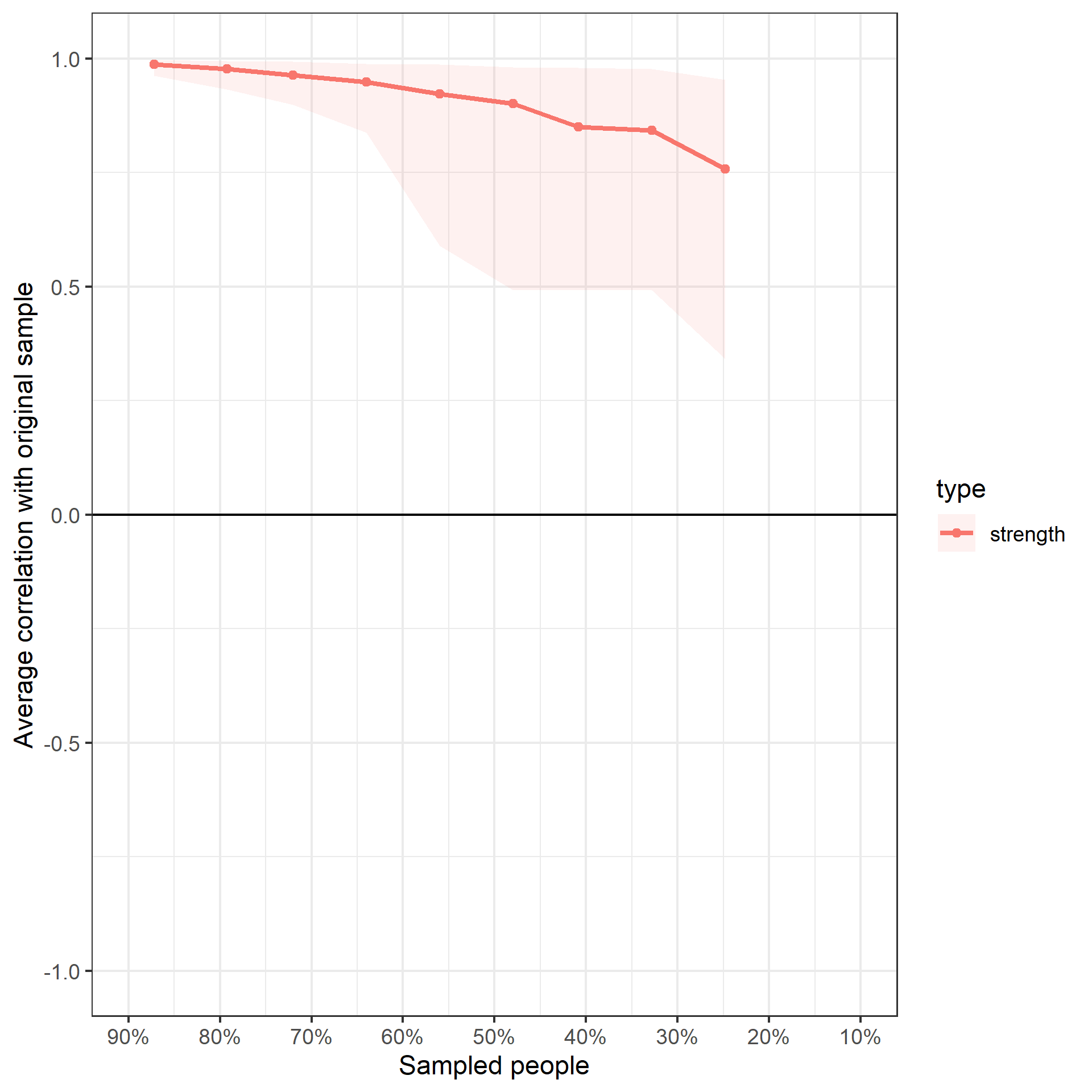


CS-coefficient: .52

1. Edge weight accuracy (left) and centrality stability (right) for model 4B: Resource network of risk group including stress indicators.


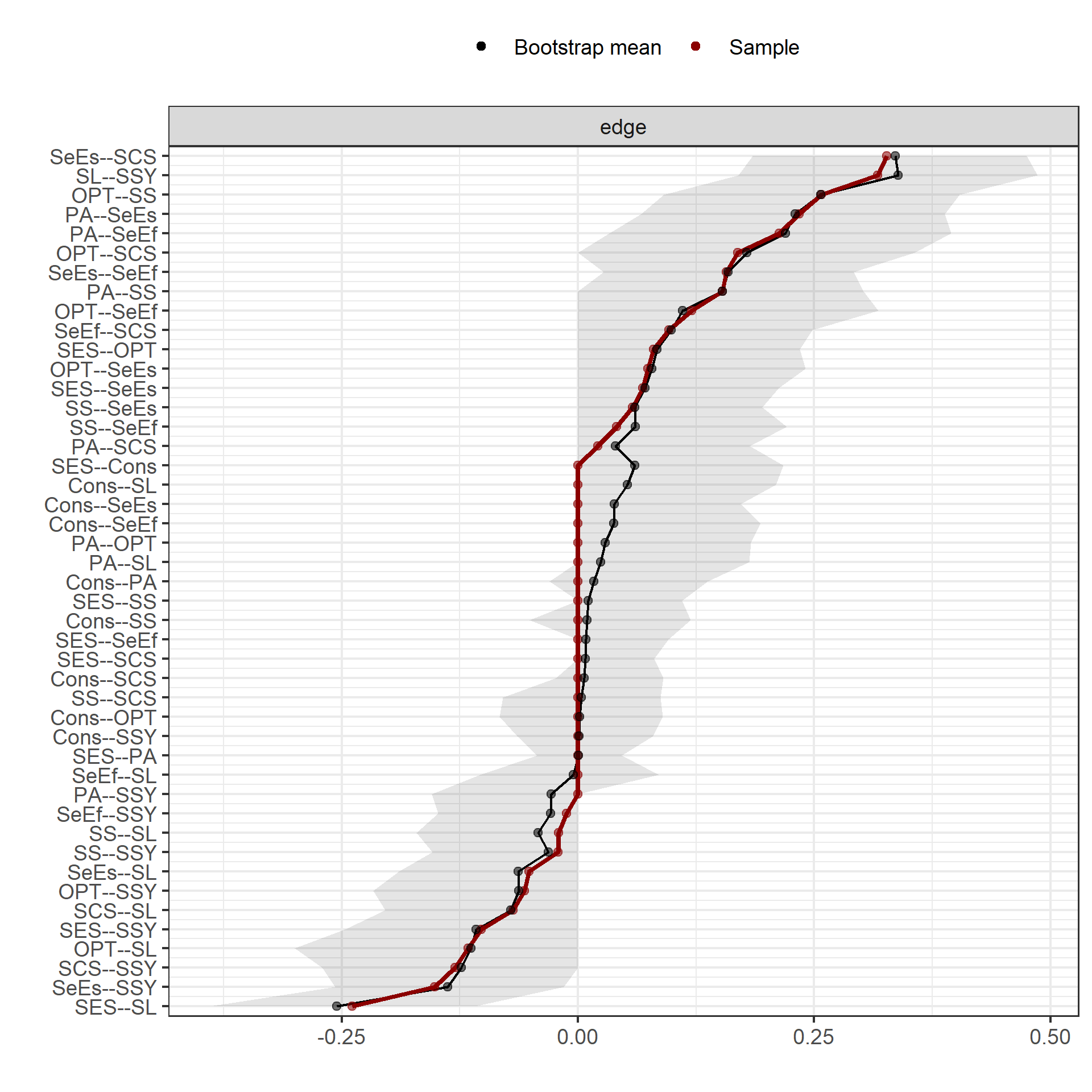

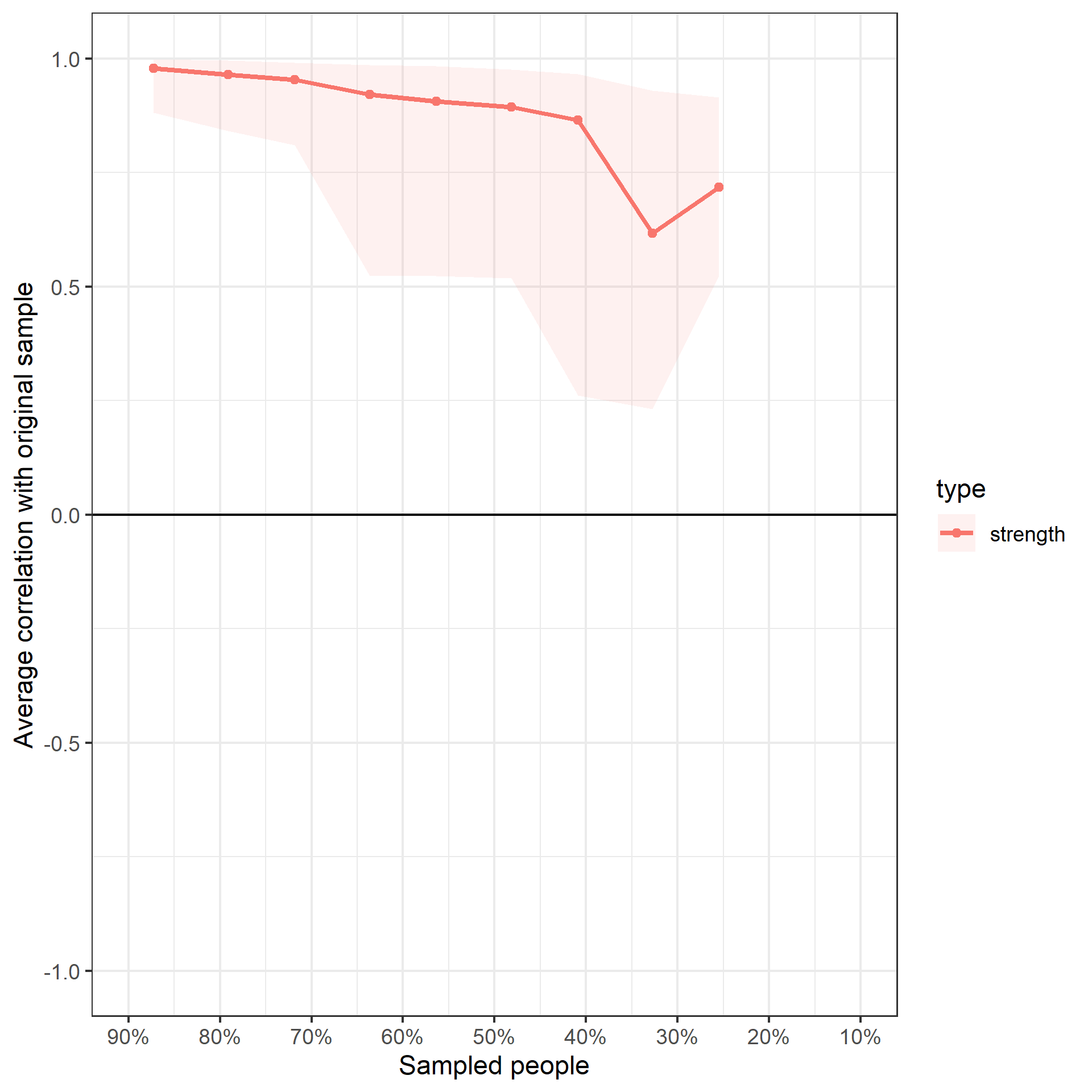


CS-coefficient: .28
